# School Refusal Assessment Scale-Revised: Factorial Invariance and Latent Means Differences across Gender and Age in Spanish Children

**DOI:** 10.3389/fpsyg.2016.02011

**Published:** 2016-12-26

**Authors:** Carolina Gonzálvez, Cándido J. Inglés, Christopher A. Kearney, María Vicent, Ricardo Sanmartín, José M. García-Fernández

**Affiliations:** ^1^Department of Developmental and Psychology and Didactics, University of AlicanteAlicante, Spain; ^2^Department of Health Psychology, Miguel Hernandez University of ElcheElche, Spain; ^3^Department of Psychology, University of Nevada, Las Vegas, Las VegasNV, USA

**Keywords:** school refusal, validation, factorial invariance, latent means differences, primary education, SRAS-R-C

## Abstract

The aim of this study was to analyze the factorial invariance and latent means differences of the Spanish version of the School Refusal Assessment Scale-Revised for Children (SRAS-R-C) in a sample of 1,078 students (50.8% boys) aged 8–11 years (*M* = 9.63, *SD* = 1.12). The results revealed that the proposed model in this study, with a structure of 18 items divided into four factors (Negative Affective, Social Aversion and/or Evaluation, To Pursue Attention and Tangible Reinforcements), was the best-fit model with a tetra-factorial structure, remaining invariant across gender and age. Analysis of latent means differences indicated that boys and 11-year-old students scored highest on the Tangible Reinforcements subscale compared with their 8- and 9-year-old peers. On the contrary, for the subscales of Social Aversion and/or Evaluation and to Pursue Attention, the differences were significant and higher in younger age groups compared to 11-year-olds. Appropriate indexes of reliability were obtained for SRAS-R-C subscales (0.70, 0.79, 0.87, and 0.72). Finally, the founded correlation coefficients of scores of the SRAS-R-C revealed a predictable pattern between school refusal and positive/negative affect and optimism/pessimism.

## Introduction

Regular class attendance is a key factor for better academic results (Yahaya et al., 2010; Thornton et al., 2013), development of social skills and conflict-resolution strategies (Kearney and Graczyk, 2014), and prevention of substance use and behavioral problems in youth (Maynard et al., 2012; Guller et al., 2015; Dembo et al., 2016; Thrul et al., 2016). School refusal, however, affects as many as 28–35% of students if the causal heterogeneity behind this behavior is considered (e.g., anxiety, pursuit of other interests outside of school hours; Mihalas, 2014). Furthermore, 28% of students in Spain have presented unjustified absences from an educational institution (Organisation for Economic Co-operation and Development, 2012). School refusal tends to affect youths aged 10–13 years, but less strong effects have been noted for gender or ethnicity (Pina et al., 2009). School refusal behavior refers to a child’s refusal to go to school and/or persistent difficulty remaining in class for an entire school day, and this behavior may or may not be based on anxiety (Hendron and Kearney, 2011). Despite high prevalence rates and the adverse consequences linked to this problem, there are limited available instruments to specifically assess this construct, especially for Spanish students (Inglés et al., 2015; García-Fernández et al., 2016). At the international level, the School Refusal Assessment Scale for Children-Revised (SRAS-R-C; Kearney, 2002a) is the most commonly used assessment scale, and it is mainly applied in American (Kearney, 2016), French (Brandibas et al., 2001, 2004), German (Hochadel et al., 2014), Korean (Kim, 2010), English (Richards and Hadwin, 2011), and Turkish (Seçer, 2014) populations. Other instruments to evaluate school refusal have been proposed to assess this construct, such as the scale Feelings of School Avoidance (Watanabe and Koishi, 2000), School Avoidance Scale (Fujigaki, 1996), and School Refusal Personality Scale (Honjo et al., 2003). However, these instruments have not been sufficiently validated.

The aim of the SRAS-R-C is to identify the four functions underlying problematic school refusal behavior: (I) To Avoid School Related Stimuli that Provoke a Sense of General Negative Affectivity, (II) To Escape from Aversive Social and/or Evaluative Situations at School, (III) To Pursue Attention from Significant Others, and (IV) To Pursue Tangible Reinforcement Outside the School Setting. After the publication of the original test (SRAS; Kearney and Silverman, 1993), several studies focused their analyses in assessing the psychometric properties of the instrument, both the initial version (Brandibas et al., 2001; Higa et al., 2002; Kim, 2010) and the revised version (Kearney, 2006; Lyon, 2010; Haight et al., 2011; Richards and Hadwin, 2011; Seçer, 2014).

To improve the psychometric quality of the original version, Kearney (2002a) revised the scale with a diverse sample of 168 youth with school refusal behavior aged 6–17 years (*M1* = 13.9, *DE* = 1.9; *M2* = 11.9, *DE* = 2.8). Results indicated adequate concurrent validity with the original version of the scale and significant scores for each of the four factors (*M* = 0.68). In addition, higher average scores in fear and trait anxiety were found in students whose school refusal was maintained by negative reinforcement. On the other hand, students whose school refusal was maintained by tangible positive reinforcement outside of the school scored higher in externalizing behaviors.

Kearney (2006) attempted to clarify and statistically determine which functional model (2, 3, or 4 factors) better adjusted with the SRAS-R-C (Kearney, 2002a). A confirmatory factor analysis (CFA) in a sample of 213 US children and adolescents with school refusal behavior aged 6–16 years revealed that none of the models met criteria for goodness-of-fit. After two items were removed from Factor IV (20 and 24), the four factor model did meet criteria for goodness-of-fit. Test-retest reliability coefficients over a period of 1–2 weeks were acceptable for each of the four factors (0.64, 0.73, 0.78, 0.56).

In addition, Lyon (2010) and Haight et al. (2011) significantly confirmed the structure of four factors with CFAs, reaching a better adjustment of the model after removing some items. On the one hand, in Lyon’s (2010) study with a community sample of 174 students aged 10–12 years, items 16, 20, and 24 were eliminated from the fourth factor. On the other hand, Haight et al. (2011) removed item 19 from the third factor and item 20 from the fourth factor with a sample of 216 students aged 11–17 years with primary school refusal behavior. Richards and Hadwin (2011) evaluated the factorial structure of the SRAS-R-C through a CFA in a community sample of 152 students aged 12–13 years. Factor IV was completely removed and a three factor structure was supported. Seçer (2014) conducted the most recent validation of the SRAS-R-C in a sample of 480 Turkish adolescents aged 13–18 years who did not refuse to go to school. This study supported the four factor structure with test-retest reliability values over a 2-week period of 0.86, and it provided a version of 19 items with the best adjustment after removing items 4, 17, 18, 19, and 20. Additional research is thus needed regarding the factorial structure of the SRAS-R-C, especially in Spanish students. There are studies that have used this scale with children aged between 5 and 9 years (Kearney et al., 2005) and 8–11 years (Hochadel et al., 2014). However, there are not previous validations of the SRAS-R-C with pre-adolescents, whereas there are different validations of this scale only with adolescents (Lyon, 2010; Haight et al., 2011). Consequently, a pre-adolescent sample participated in this study in order to avoid analyzing this behavior in a wide age range covering late childhood and adolescence. The aim of this study was to validate the SRAS-R-C in a representative sample of Spanish students aged 8–11 years. This general aim can be specified in the following statements: (1) To examine the factorial invariance of the SRAS-R-C across gender and age groups; (2) To analyze the latent means differences across gender and age; (3) To examine the correlations between school refusal and positive/negative affectivity and optimism/pessimism.

## Materials and Methods

### Ethics Statement

This research was reviewed and approved by the Directorate-General of Scientific and Technological Research-Ministry of Economy and Competitiveness (Spain) (EDU2012-35124) and according to the standards established by the Ethics Committee of the University of Alicante and the University Miguel Hernandez.

### Participants

The initial sample of this study comprised 1416 students recruited by random cluster sampling in four Spanish provinces (Alicante, Albacete, Murcia, and Sevilla). Four school centers were selected from each province. As a result, 16 school centers were selected, which are disaggregated in 12 public centers, two state-funded schools and two private schools, located in urban and rural zones. Once the school centers were determined, a class for each academic year was randomly selected (four classes per center), and on average each school contributed 89 participants. From the initial sample, 182 students (12.85%) were excluded because they did not obtain paternal consent to participate in the research, 108 (7.63%) because of omissions and mistakes in their answers, and 48 (3.39%) due to the fact that they were non-Spanish students. The final sample included 1078 participants, 548 boys and 530 girls (50.8 and 49.2%, respectively), aged 8–12 years (*M* = 9.63; *SD* = 1.12). No statistically significant differences between the four groups were found with respect to gender and academic year.

### Measures

The School Refusal Assessment Scale Revised for Children (SRAS-R-C; Kearney, 2002a) is the validated instrument in this study and it is a scale with good psychometric properties that were presented earlier.

The Positive and Negative Affect Schedule for Children (10-Item PANAS-C; Ebesutani et al., 2012) is a 10-item self-report measure of positive and negative affect in children and young people aged 6–18 years. The subscales have demonstrated appropriate rates of internal consistency (0.86 positive affect and 0.82 negative affect; Ebesutani et al., 2012). Both subscales were used for this study and the coefficients of internal consistency (Cronbach’s alpha) were 0.75 for the positive affect and 0.76 for the negative affect.

The Youth Life Orientation Test (YLOT; Ey et al., 2005) is a 16-item measure of positive expectations (optimism) and negative expectations (pessimism). The subscales have demonstrated an acceptable range for internal consistency and have shown high 1-month test-retest reliability (0.68 for optimism and pessimism; Ey et al., 2005). Convergent and discriminant validity were demonstrated via association with other measures of hope and self-efficacy. Both subscales were used in this study and the coefficients of internal consistency (Cronbach’s alpha) were 0.79 for optimism and 0.78 for pessimism.

The same translation process was conducted for the three scales used in this study (Hambleton and Lee, 2015). First, the items in the original versions in English were translated to Spanish by a native expert in educational psychology and with university studies in English translation. Once the translation was finished, a native English speaker with Spanish knowledge and an expert in psychology performed the back-translation to the original language. Finally, we evaluated and compared again the adapted version by a group of judges-experts endorsing proper translation.

### Procedure

To start, a meeting with the principals and the education center management team was held to expose the objectives of this research work and the evaluation instruments used, to ask for their permission, and to achieve their collaboration. Afterward, an informative letter was written to inform the students’ parents about the purpose of this study and to ask for their written consent to allow their children to participate in the research activity.

The measuring instruments were administered to the whole class as a collective and its completion was voluntary, assigning a key to the test battery for each of the participants. During the fulfillment of the questionnaires, the researcher was there to solve doubts, and the tutor of the different academic years also provided assistance. Each of the classes was composed by 20 students and one session of 50 min was used to complete the tests (15–20 min the SRAS-R-C, 5–10 min the PANAS-C, and 10–15 min the YLOT).

### Statistical Analyses

To examine the different proposed model for the SRAS-R-C, nine confirmatory factor analyses (CFA) were conducted over nine alternative proposals based on previous research and an own model designed in this investigation. The polychoric correlation matrix was analyzed using weighted least squares (WLS) to confirm the parameters of the different models. It was confirmed that there was no multivariate normality. Thus, the Mardia’s coefficient was 93.43 because a value of five points is considered as the limit for a multivariate normal distribution (Bentler, 2005). Therefore, the Satorra–Bentler scaled χ^2^ (S–Bχ^2^) was used. In addition, different goodness of fit indices were applied to verify the adequacy of the models on the factor structure of the SRAS-R-C. The next goodness-of-fit indexes were calculated: the Robust Root Mean Square Error of Approximation (R-RMSEA: <0.08 acceptable and <0.06 excellent), the Standardized Root Mean Square Residual (SRMR: close to 0.08 acceptable and <0.05 good fit), the Robust Comparative Fit Index (R-CFI: ≥0.90 acceptable and >0.95 good fit), and the Tucker Lewis Index (TLI: ≥ 0.90 acceptable; Hu and Bentler, 1999; Brown, 2006). In addition, a classic item analysis was performed and the reliability of the scale and its factors were checked by Cronbach’s alpha coefficient, whose α values were considered acceptable at ≥0.70.

To examine the factor invariance of the SRAS-R-C, for the four factor model proposed in this study, a multigroup CFA was performed to check the invariance of the measurement and structure through groups of gender and age. To do this, several hierarchical steps were followed based on the existing literature (Byrne, 2008a,b; Liu et al., 2015; Samuel et al., 2015). To check the good fit of the models, the goodness-of-fit indexes explained before were used (R-RMSEA, SRMR, R-CFI, and TLI) along with the criteria of invariance: ΔCFI (ΔCFI < 0.01; Cheung and Rensvold, 2002) and the level of non-significant probability associated to the ΔS–Bχ^2^ (Dimitrov, 2010). Finally, the latent means difference across gender and age were performed by the Critical Ratio (CR), considering that with results >1.96 or <-1.96 the estimation of equality is rejected (Tsaousis and Kazi, 2013). Besides, effect sizes were measured using Cohen’s *d* (Fritz et al., 2012). Regarding the gender, the group of girls was set to zero to develop its comparison with the boys. About the age, each group was successively set to zero to establish the comparative with other groups freely estimated.

The correlations between the different factors of the SRAS-R-C were obtained by Pearson product-moment correlation coefficient, as well as its discriminant validity from measures that evaluate the affect and optimism/pessimism. The interpretation of these results was performed according to the criteria proposed by Cohen (1988) about the magnitude of the effect sizes. Statistical analyzes were calculated using SPSS 20 and EQS 6.1 statistical programs.

## Results

### Confirmatory Factor Analyses

Data regarding the CFAs are in **Table 1**. Six of the tested models supported the four factor structure (Models 4a, 4b, 4c, 4cII, 4d, and 4e), whereas the remaining two models showed a better adjustment with a three factor structure (Models 3a and 3b). Model 3b with 12 items was the model with the best-fit indices. However, if four factor models are considered, then the model proposed by the research group showed the best goodness-of-fit indices. As a result, a final four factor structure formed by 18 items is proposed for the Spanish version of the SRAS-R-C: Factor I. Avoid School Related Stimuli that Provoke Negative Affectivity (1, 5, 9, 13, 21), Factor II. Escape Aversive Social and/or Evaluative Situations at School (2, 6, 10, 14, 22), Factor III. Pursue Attention from Significant Others (3, 7, 11, 15, 23), and Factor IV. Pursue Tangible Reinforcement Outside the School Setting (4, 8, 12).

**Table 1:** Confirmatory factor analyses: goodness-of-fit indices of the statistic models of the School Refusal Assessment Scale-Revised for Children (SRAS-R-C).

	S–Bχ^2^	*df*	R-RMSEA 90% CI	SRMR	R-CFI	TLI
Model 3a	1560.95	249	0.070 [0.067–0.073]	0.087	0.675	0.640
Model 3b	237.67	51	0.040 [0.032–0.048]	0.045	0.931	0.911
Model 4a	1261.53	246	0.062 [0.058–0.065]	0.074	0.748	0.718
Model 4b	676.85	183	0.050 [0.046–0.054]	0.073	0.841	0.818
Model 4c	692.28	183	0.051 [0.047–0.055]	0.059	0.839	0.816
Model 4cII	530.63	182	0.042 [0.038–0.046]	0.053	0.890	0.873
Model 4d	1015.83	203	0.061 [0.057–0.065]	0.073	0.767	0.735
Model 4e	482.36	146	0.046 [0.042–0.051]	0.055	0.877	0.856
Own model	341.7284	129	0.039 [0.034–0.044]	0.050	0.917	0.901


*Model 3a: Kearney (2002b); Model 3b: Richards and Hadwin (2011); Model 4a: Kearney (2002b); Model 4b: Kearney (2006); Model 4c: Lyon (2010); Model 4cII: Lyon (2010) items 17–18 correlated; Model 4d: Haight et al. (2011); Model 4e: Seçer (2014); S–Bχ^2^, Satorra–Bentler scaled χ^2^; *df*, degrees of freedom; R-RMSEA, Robust Root Mean Square Error of Approximation; CI, confidence interval; SRMR, Standardized Root Mean Square Residual; R-CFI, Robust Comparative Fit Index; TLI, Tucker Lewis Index. *p* < 0.001 for S–Bχ^2^ in all cases.*

Cronbach reliability coefficients for each of the four factors were 0.70 (I. Avoid School Related Stimuli that Provoke Negative Affectivity), 0.79 (II. Escape Aversive Social and/or Evaluative Situations at School), 0.87 (III. Pursue Attention from Significant Others), and 0.72 (IV. Pursue Tangible Reinforcement Outside the School Setting). The 2-week test-retest reliability ranged from 0.70 (IV. Pursue Tangible Reinforcement Outside the School Setting) to 0.75 (II. Escape Aversive Social and/or Evaluative Situations at School).

### Classical Item Analysis

Item means ranged from 4.53 (item 8) to 0.41 (item 6) and the standard deviation ranged from 2.21 (item 23) to 1.06 (item 6). The item-scale correlation coefficients for the four factors ranged from 0.63 (III. Pursue Attention from Significant Others. Items: 2, 6, and 22) to 0.81 (IV. Pursue Tangible Reinforcement Outside the School Setting. Item: 12). Internal consistency of the items with one item removed ranged from 0.75 (III. Pursue Attention from Significant Others. Item: 11) to 0.79 (IV. Pursue Tangible Reinforcement Outside the School Setting. Item: 8).

### Factorial Invariance across Gender and Age for the SRAS-R-C

Results regarding measurement and structural invariance across gender and age are in **Tables 2** and **3**, respectively. A stepwise hierarchical method was applied to analyze the invariance of the SRAS-R-C in which restrictions were imposed on the obtained model. First, a baseline model (Model 0) with no equality constraints across gender or age showed adequate goodness-of-fit indices (TLI and R-CFI > 0.90; R-RMSEA < 0.05; SRMR < 0.08; ΔCFI < 0.01). All factor loadings were then constrained to be equal across gender and age groups to calculate the metric invariance (Model 1). The goodness-of-fit indices for this model were also reasonable. Subsequently, constraints were set on the intercepts of the variables and a new model was created (Model 2), which showed adequate goodness-of-fit indices. Then, to establish measurement invariance, strict invariance (Model 3) set the factor loadings, the intercepts of the variables, and the variances and covariances of the errors. This model also showed reasonable goodness-of-fit indices. Finally, the structural invariance (Model 4) consisted of setting the variances of the factors and equalizing their covariances in Model 2. The goodness-of-fit indices of this nested model also were adequate. Moreover, the Satorra–Bentler scaled χ^2^ was used in all the tested models (Models 1, 2, 3, and 4) in gender and age. All ΔS–Bχ^2^ of the tested models showed no statistically significant differences (*p* > 0.05), so the measurement and structural invariance of the SRAS-R-C were confirmed across gender and age.

**Table 2:** Goodness-of-fit indices for the own model of the SRAS-R-C depending on gender.

	χ^2^	S–Bχ^2^	*df*	TLI	R-CFI	R-RMSEA	SRMR	ΔS–Bχ^2^ (Δ*df*, *p)*	ΔCFI
Boys	348.54	256.65	129	0.901	0.915	0.042 [0.035–0.050]	0.051		
Girls	301.28	217.77	129	0.920	0.934	0.036 [0.028–0.044]	0.052		
Model 0	649.82	474.06	258	0.910	0.924	0.028 [0.024–0.032]	0.052		
Model 1	667.53	482.23	272	0.918	0.926	0.027 [0.023–0.031]	0.054	10.84 (14, 0.698)	0.002
Model 2	684.90	500.99	290	0.913	0.925	0.027 [0.023–0.030]	0.054	15.69 (18, 0.614)	-0.001
Model 3	717.86	505.91	308	0.917	0.928	0.025 [0.022–0.029]	0.055	14.63 (18, 0.687)	0.003
Model 4	697.23	507.02	300	0.918	0.927	0.026 [0.022–0.030]	0.057	7.66 (10, 0.662)	0.002


*Model 0 = Free model; Model 1 = Model 0 with factor loadings; Model 2 = Model 1 with intercepts; Model 3 = Model 2 with error variances; Model 4 = Model 2 with variances and covariance factors; S–Bχ^2^ = Satorra–Bentler χ^2^ scaled; *df*, degrees of freedom; TLI, Tucker-Lewis Index; R-CFI, Robust Comparative Fit Index; R-RMSEA, Robust Root Mean Square Error of Approximation; SRMR, Standardized Root Mean Square Residual; ΔCFI, comparative fit index difference test. ΔS–Bχ^2^ = χ^2^ difference model comparison test; Δ*df*, difference between degrees of freedom.*

**Table 3:** Goodness-of-fit indices for the own model of the SRAS-R-C depending on age.

	χ^2^	S–Bχ^2^	*df*	TLI	R-CFI	R-RMSEA	SRMR	ΔS–Bχ^2^ (Δ*df*, *p*)	ΔCFI
8 year old	195.72	157.38	129	0.933	0.943	0.032 [0.005–0.047]	0.058		
9 year old	207.11	147.29	129	0.967	0.972	0.022 [0.000–0.038]	0.058		
10 year old	257.82	192.28	129	0.920	0.934	0.044 [0.031–0.057]	0.060		
11 year old	294.35	222.70	129	0.900	0.918	0.054 [0.042–0.066]	0.066		
Model 0	954.99	719.11	516	0.930	0.942	0.020 [0.016–0.023]	0.060		
Model 1	1046.73	767.81	558	0.925	0.939	0.019 [0.016–0.023]	0.069	51.05 (42, 0.160)	-0.003
Model 2	1095.61	820.35	612	0.914	0.934	0.019 [0.015–0.022]	0.069	46.63 (54, 0.751)	-0.005
Model 3	1251.24	874.49	666	0.911	0.932	0.019 [0.015–0.022]	0.072	61.98 (54, 0.213)	-0.002
Model 4	1178.33	849.16	642	0.916	0.932	0.019 [0.015–0.022]	0.079	33.76 (30, 0.291)	-0.002


*Model 0 = Free model; Model 1 = Model 0 with factor loadings; Model 2 = Model 1 with intercepts; Model 3 = Model 2 with error variances; Model 4 = Model 2 with variances and covariance factors; S–Bχ^2^, Satorra–Bentler χ^2^ scaled; *df*, degrees of freedom; TLI, Tucker-Lewis Index; R-CFI, Robust Comparative Fit Index; R-RMSEA, Robust Root Mean Square Error of Approximation; SRMR, Standardized Root Mean Square Residual; ΔCFI, comparative fit index difference test; Δ–Bχ^2^ = χ^2^ difference model comparison test; Δ*df*, difference between degrees of freedom.*

### Latent Means Differences across Gender and Age on the SRAS-R-C

The model for comparing gender groups used girls as the reference group. Given that there were four age groups (8, 9, 10, and 11 years), three reference models were established to make all possible comparisons. In each model, the lowest age group was set to zero: Model 1 = 8- versus 9-, 10-, and 11-year-olds; Model 2 = 9- versus 10- and 11-year-olds; and Model 3 = 10- versus 11-year-olds. For gender groups, the fit statistics of the latent mean structures were reasonable (χ^2^ = 678.23, *df* = 286, *p* < 0.000, R-CFI = 0.906, R-RMSEA = 0.036, CI = 0.033–0.040, and SRMR = 0.054). Fit statistics of the latent mean structures for the age groups were reasonable in all cases (Model 1: χ^2^ = 1078.24, ?*df* = 600, *p* < 0.000, R-CFI = 0.917, R-RMSEA = 0.029, CI = 0.026–0.032, and SRMR = 0.072; Model 2: χ^2^ = 837.93, *df* = 495, *p* < 0.000, R-CFI = 0.915, R-RMSEA = 0.035, CI = 0.031–0.038, and SRMR = 0.072; Model 3: χ^2^ = 608.44, *df* = 286, *p* < 0.000, R-CFI = 0.906, R-RMSEA = 0.048, CI = 0.043–0.053, and SRMR = 0.074).

The structured mean differences across gender and age groups are in **Table 4**. With regard to gender, no statistically significant differences were found except for Factor IV (Pursue Tangible Reinforcement Outside the School Setting), in which boys had significantly higher structured means than girls with a small size effect size (*d* = 0.13). With respect to age groups, 11-year-olds had significantly higher means in Factor IV than 8- (*d* = 2.13) and 9-year-olds (*d* = 2.07). However, 11-year-olds had significantly lower means in the second factor (To Escape Aversive Social and/or Evaluative Situations at School) than 8-year-olds (*d* = -0.19) and in the third factor (To Pursue Attention from Significant Others) than 8- (*d* = -0.37), 9- (*d* = -0.27), and 10-year olds (*d* = -0.38). The effect sizes were small except for the fourth factor that presented a large effect size. No statistically significant differences were found on the scores of the first factor of the SRAS-R-C (To Avoid School Related Stimuli that Provoke Negative Affectivity) across gender.

**Table 4:** La means differences across gender and age groups in the SRAS-R-C.

	SRAS-R-C factors			
	
	FI	FII	FIII	FIV
**Girls (reference)**				
**Boys**				
Mean estimate (ME)	0.07	0.03	0.27	0.24
Standard error (SE)	0.06	0.05	0.19	0.12
Critical Ratio (CR)	1.14	0.59	1.40	2.08
**8-year-old (reference)**				
**9-year-old**				
ME	0.07	-0.05	-0.23	-0.08
SE	0.08	0.07	0.16	0.13
CR	0.79	-0.72	-1.44	0.62
**10-year-old**				
ME	0.16	-0.03	-0.14	0.08
SE	0.09	0.08	0.16	0.14
CR	1.75	-0.41	-0.91	0.62
**11-year-old**				
ME	0.15	-0.16	-0.76	1.95
SE	0.09	0.08	0.19	0.12
CR	1.70	-1.99	-3.93	15.77
**9-year-old (reference)**				
**10-year-old**				
ME	0.08	0.02	0.10	0.04
SE	0.07	0.07	0.15	0.14
CR	1.10	0.21	0.71	0.27
**11-year-old**				
ME	0.10	-0.09	-0.42	1.95
SE	0.07	0.07	0.14	0.01
CR	1.41	-1.31	-3.04	16.53
**10-year-old (reference)**				
**11-year-old**				
ME	-0.05	-0.14	-0.56	0.22
SE	0.08	0.08	0.14	0.14
CR	-0.68	-1.79	-3.97	1.50


*FI, Negative Affectivity; FII, Social Aversion and/or Evaluation; FIII, To Pursue Attention; FIV, To Pursue Tangible Reinforcement.*

### Correlation Coefficients between the Factors of the SRAS-R-C

The correlation coefficients between the different factors of the SRAS-R-C and the total score of the scale were statistically significant (**Table 5**). With regard to the correlation coefficients between the four factors of the SRAS-R-C, the first two factors showed a statistically significant and positive correlation of high magnitude (0.53), whereas the first and third factor (0.34) and the second and third factor (0.37) showed moderate correlations. Finally, the fourth factor showed a statistically significant and positive but low correlation (0.21) with the third factor. The two first factors of the SRAS-R-C thus did not correlate with the fourth factor, as expected.

**Table 5:** Correlations between factors and the total score of SRAS-R-C.

SRAS-R-C factors	Total SRAS-R-C	FI	FII	FIII
FI. To Avoid School Related Stimuli that Provoke a Sense of General Negative Affectivity.	0.70^∗^			
FII. To Escape from Aversive Social and/or Evaluative Situations at School.	0.67^∗^	0.53^∗^		
FIII. To Pursue Attention from Significant Others.	0.81^∗^	0.34^∗^	0.37^∗^	
FIV. To Pursue Tangible Reinforcement Outside the School Setting.	0.41^∗^	0.01	-0.05	0.21^∗^


*^∗^Correlation is significative *p* < 0.001.*

### Correlation Coefficients between the SRAS-R-C and the PANAS and YLOT

Correlation coefficients of the subscales and the total score of the SRAS-R-C with the PANAS and the YLOT are in **Table 6**. The first three factors and the total score of the SRAS-R-C were positively correlated with negative affect and pessimism, whereas the fourth factor of the SRAS-R-C was positively correlated with positive affect and optimism. In addition, the total score of the SRAS-R-C was inversely correlated (-0.12) with the PANAS-C positive affect subscale and positively correlated (0.32) with the PANAS-C negative affect subscale. Finally, the total score of the SRAS-R-C was positively correlated (0.23) with the YLOT subscale of pessimism. No statistically significant correlation was found with respect to the YLOT subscale of optimism.

**Table 6:** Concurrent validity.

		FI	FII	FIII	FIV	Total
	Factors	SRAS-R-C	SRAS-R-C	SRAS-R-C	SRAS-R-C	SRAS-R-C
PANAS-C	Positive affect	-0.24^∗∗^	-0.21^∗∗^	n.s.	0.18^∗∗^	-0.12^∗∗^
	Negative affect	0.40^∗∗^	0.41^∗∗^	0.16^∗∗^	n.s.	0.32^∗∗^
YLOT	Optimism	-0.17^∗∗^	-0.18^∗∗^	n.s.	0.19^∗∗^	n.s.
	Pessimism	0.32^∗∗^	0.33^∗∗^	0.11^∗∗^	-0.15^∗∗^	0.23^∗∗^


*SRAS-R-C, School Refusal Assessment Scale-Revised for Children; PANAS-C, The 10-Item Positive and Negative Affect Schedule for Children; YLOT, Youth Life Orientation Test. ^∗∗^*p* < 0.01.*

## Discussion

The aim of this study was to validate an instrument to assess school refusal in Spanish sample in terms of the causes that determine this behavior. At the present time, the validation of this instrument has been completed in other countries such as United Kingdom (Richards and Hadwin, 2011) and Turkey (Seçer, 2014). In this sense, the current investigation is the first study to offer the validation of the SRAS-R-C in Spanish language. The proposed model of this work, with a structure of 18 items divided into four factors, was the best-fit model with a tetra-factorial structure remaining invariant across gender and age. Latent means differences indicated that boys and 11-year-olds scored higher on the Tangible Reinforcements subscale compared with girls and their 8- and 9-year-old peers, respectively. On the contrary, for the subscales of Social Aversion and/or Evaluation and to Pursue Attention, the differences were significant and higher in younger age groups compared to 11-year-olds. The magnitude of the differences found was small in all cases except for the fourth factor. Concretely, the oldest group of students (11-year-olds) reported higher scores on the fourth factor in comparison with younger students. Appropriate indexes of reliability were obtained for the SRAS-R-C and the correlation coefficients revealed a predictable pattern between school refusal and positive/negative affect and optimism/pessimism. These results are discussed below.

Taking into consideration the different goodness-of-fit indices conducted in this study, the model proposed by Richards and Hadwin (2011) obtained the best adjustment with a three factor structure. Nonetheless, this proposal has a strong limitation because it completely removes the fourth factor. The model proposed in the present study of 18 items divided into four factors was the instrument with the best adjustment to evaluate school refusal from a broad perspective that includes the different factors that can cause this type of behavior. These results also support the four factor structure of the SRAS-R-C according to the most previous research (Kearney and Silverman, 1993; Kearney, 2006; Lyon, 2010; Haight et al., 2011; Seçer, 2014). In the own model, items 16, 17, 18, 19, and 20 were omitted. The removal of items 17 (Factor I: *If you had less bad feelings (e.g., scared, nervous, sad) about school, would it be easier for you to go to school?*) and 18 (Factor II: *If it were easier for you to make new friends, would it be easier for you to go to school?*) is in line with the findings of Seçer (2014), who argued that these items were written in conditional style and complicated the understanding of the scale. Item 19 (Factor III: *Would it be easier for you to go to school if your parents went with you?*) was removed from the scale because it did not reach satisfactory performance, similar to Haight et al. (2011) and Seçer (2014). As Lyon (2010) mentioned, the hypothetical approach of these items might be confusing. Finally, items 16 (*How often do you refuse to go to school because you want to have fun outside of school?*), 20 [*Would it be easier for you to go to school if you could do more things you like to do after school hours (e.g., being with friends)?*], and 24 (*Would you rather be doing fun things outside of school more than most kids your age?*) were removed from Factor IV in accordance with Lyon (2010). Its removal improved model fit due to the fact that item 16 was the least specific of the scale (Lyon, 2010), whereas items 20 and 24 established a comparative with the rest of the items and their interpretation might be misinterpreted. Both items were also omitted in Kearney (2006).

The obtained results revealed reasonable indices of internal consistency, goodness-of-fit, and temporal stability for the four factors of the SRAS-R-C. Concerning the proposed classification by George and Mallery (2003), the obtained values were acceptable and more adjusted than the results obtained by more recent validations (Haight et al., 2011; Seçer, 2014). Moreover, the multigroup CFA supported the measurement and structure invariance of the scale across gender and age, so the third hypothesis was confirmed.

The present study revealed measurement and structure invariance across gender and age of the Spanish version. These results enhance the use of this version to assess latent means differences across gender and age on school refusal. Factor IV was the only one that obtained statistically significant gender differences. In this case, boys scored significantly higher than girls, which is in line with studies that indicate a greater prevalence of males in Factor IV or truancy not based on anxiety (Kearney, 2008; Kearney and Spear, 2014). Regarding age, Factors II, III, and IV presented statistically significant differences. The later age group obtained significantly higher Factor IV means with a large size magnitude in comparison with 8- and 9-year-olds. Several studies explained that high scores in this factor are more frequent during adolescence rather than childhood (Inglés et al., 2015; Kearney, 2016). In addition, these findings might be justified by the fact that this kind of school refusal is connected with truancy, which is a common problem in students of later ages (Kearney and Albano, 2004).

With respect to Factor II of the SRAS-R-C, it was not expected to obtain statistically higher means in 8-year-olds in comparison with 11-year-olds because later age students have obtained the highest scores in this factor (Kearney and Albano, 2004). However, it is important to consider that this factor evaluates two different aspects of the same construct. On the one hand, there are items of Factor II that assess school avoidance due to reasons of social aversion, and adolescence is a time when students worry about social acceptability (Puklek et al., 2015; Thomas et al., 2015). On the other hand, the second dimension of Factor II assesses anxiety caused by academic evaluation and, in this point, 8-year-olds in Spain experience a significant increase of exigency due to the diagnostic test that is scheduled at the end of the academic year (LOMCE).

With respect to Factor III, Kearney and Albano (2004) associated this factor with pursuing attention from significant others and early school ages. Moreover, other studies highlight the relation between Factor III and separation anxiety disorder, which is more prevalent in young children (Kearney and Silverman, 1993; Higa et al., 2002; Kearney, 2002a). In this way, it might be justified that 11-year-olds obtained significantly lower scores in Factor III in comparison with the early age groups. At this point, it is important to expand empirical research about latent means differences across gender and age to contrast the obtained results with both international and national samples.

Although this study supports the four factor structure of the SRAS-R-C, the results revealed the existence of correlations between the three first factors and between Factors III and IV, which were of a high magnitude between the two first factors (0.53) and of low magnitude between Factors III and IV (0.21). These findings are in accordance with Kearney and Silverman (1993) and Higa et al. (2002). In this line, the results verify that there is no correlation between the two first factors and the fourth factor of the SRAS-R-C (Kearney and Silverman, 1993; Higa et al., 2002). This fact might be justified because the three first factors of the SRAS-R-C base school refusal on anxiety symptoms caused by the school in general (Factor I), caused by social or evaluative situations inside the school environment (Factor II), or caused by the separation of a closed person (Factor III). In contrast, Factor IV school refusal is based on obtaining tangible reinforcements outside the school setting, such as staying home playing videogames or to be with friends during school hours (Kearney, 2008).

The study of interaction between school refusal and affect, optimism, and pessimism has lacked empirical evidence. The negative affect dimension of the PANAS-C significantly and negatively correlated with the three first factors of the SRAS-R-C and the total score of the scale. Negative affect has been associated with feelings of sadness, fear, blame, and anger (Ebesutani et al., 2012; Pires et al., 2013), or even anxiety and depression (Watson et al., 1988; Chorpita and Daleiden, 2002; Robles and Páez, 2003; Inglés et al., 2016). Several studies have obtained positive correlations of these variables with the first three factors of the SRAS-R-C (Kearney and Silverman, 1993; Higa et al., 2002). Considering the tripartite model of anxiety, depression, and affect (Joiner et al., 1996; Sandín et al., 1999), these positive correlations were expected. These correlations provide preliminary support for the convergent and discriminant validity of the SRAS-R-C because negative affect positively correlated with the first three factors of the SRAS-R-C and the total score of the scale, whereas positive affect positively correlated with Factor IV of the SRAS-R-C, which does not justify school refusal with anxiety.

Finally, the YLOT dimension of pessimism positively and significantly correlated with the three first factors of the SRAS-R-C and the total score of the scale. These results are supported by Gisbert-Ferrándiz et al. (2013) in a simple of 342 Spanish students of primary education, which is the only study that specifically evaluated the correlation between these variables. In their study, the positive and significant correlations between pessimism and the first three factors of the SRAS-R-C predominated, whereas Factor IV positively and significantly correlated with optimism.

## Limitations and Practical Implications

The current study presents certain limitations that might be solved in future investigations. On the one hand, it might be convenient to analyze variations of school refusal behavior over time with longitudinal studies, which are insufficient in current research (Kearney, 2008) and to examine the reliability and validity of the scale in higher levels of education with a wider age range of students. Moreover, to not only consider the students’ perceptions, it might be interesting for future research to collect parent and teachers reports and to compare the scale in school refusing versus non-school refusing populations to assess its predictive validity. Finally, future works should evaluate in depth the relation of the SRAS-R-C with other instruments, as well as to validate the version addressed to parents and teachers as Kearney (2006) suggested. School refusal behavior should be related with other variables that would reinforce the knowledge of this construct, such as school anxiety, academic self-attributions, personality traits, and health perception.

The findings of this study are useful at a pragmatic level in prevention and evaluation of this problem and in its treatment. With regard to the assessment, this work offers the first Spanish validation of the SRAS-R-C in primary education and it provides the main international instrument to specifically assess school refusal for psychology and education. In this line, early detection will be encouraged as a prevention mechanism because several studies have suggested that the progressive development of school refusal could be chronic school absenteeism or school dropout (Kearney, 2008). This is important because Spain has the highest percentage of early school dropouts (21.9%) in comparison with the rest of countries of the European Union (Ministry of Education, Culture and Sport, 2015). Being able to identify school refusal behaviors during the early stages of education, such as primary education, would enable an early intervention and develop actions to overcome difficulties or negative ideas associated with the educational center. Additionally, the results of this study using latent means differences analysis have confirmed the complexity of this problem and that the factors of school refusal do not affect in the same measure across gender and age. Consequently, it is important to value with different attention those groups at greatest risk. This study offers a prevention tool and interesting data for intervention.

## Author Contributions

CG has participated conducting a literature review and writing this manuscript. CI has made substantial contributions to the conception and design of this research. He has also received the study in all its phases. CK has participated drafting the work and revising it critically for important intellectual content in all its phases. MV has participated conducting a literature review and writing this manuscript. RS has participated performing statistical analyses. JG-F has designed this research and participated performing statistical analyses.

## Conflict of Interest Statement

The authors declare that the research was conducted in the absence of any commercial or financial relationships that could be construed as a potential conflict of interest.
